# Elevation and landscape change drive the distribution of a montane, endemic grassland bird

**DOI:** 10.1002/ece3.6500

**Published:** 2020-07-06

**Authors:** Abhimanyu Lele, M. Arasumani, C. K. Vishnudas, Viral Joshi, Devcharan Jathanna, V. V. Robin

**Affiliations:** ^1^ Indian Institute of Science Education and Research Tirupati Tirupati Andhra Pradesh India; ^2^ Hume Centre for Ecology and Wildlife Biology Kalpetta Kerala India; ^3^ The Gandhigram Rural Institute Gandhigram Tamil Nadu India; ^4^ Wildlife Conservation Society – India Bengaluru Karnataka India; ^5^ Present address: Committee on Evolutionary Biology University of Chicago Chicago Illinois USA; ^6^ Present address: Negaunee Integrative Research Center Field Museum of Natural History Chicago Illinois USA

**Keywords:** *Acacia*, conservation, habitat heterogeneity, occupancy, shola ecosystem, shola sky islands, tropical montane ecosystems

## Abstract

**Context:**

Tropical montane habitats support high biodiversity and are hotspots of endemism, with grasslands being integral components of many such landscapes. The montane grasslands of the Western Ghats have seen extensive land‐use change over anthropogenic timescales. The factors influencing the ability of grassland‐dependent species to persist in habitats experiencing loss and fragmentation, particularly in montane grasslands, are poorly known.

**Objectives:**

We studied the relationship between the Nilgiri pipit *Anthus nilghiriensis*, a threatened endemic bird that typifies these montane grasslands, and its habitat, across most of its global distribution. We examined what habitat features make remnant grasslands viable, which is necessary for their effective management.

**Methods:**

We conducted 663 surveys in 170 sites and used both single‐season occupancy modeling and *N*‐mixture modeling to account for processes influencing detection, presence, and abundance.

**Results:**

Elevation had a positive influence on species presence, patch size had a moderate positive influence, and patch isolation had a moderate negative influence. Species abundance was positively influenced by elevation and characteristics related to habitat structure, and negatively influenced by the presence of invasive woody vegetation.

**Conclusions:**

The strong effect of elevation on the highly range‐restricted Nilgiri pipit is likely to make it vulnerable to climate change. This highly range‐restricted species is locally extinct at several locations, and persists at low densities in remnants of its habitat left by recent fragmentation. Our findings indicate a need to control and reverse the spread of exotic woody invasives to preserve the grasslands themselves and the specialist species dependent upon them.

## Graphical abstract

The montane grassland ecosystem of the Indian Western Ghats is experiencing severe anthropogenic threats. We examined factors influencing the occupancy and abundance of the Nilgiri pipit, endemic to these grasslands. We found that the presence and abundance of the species are strongly shaped by elevation, making it vulnerable to climate change, and is also shaped by landscape change, including invasive woody invasion, which has driven substantial local extinction.

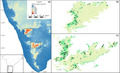

## 1 INTRODUCTION

Tropical montane habitats are highly biodiverse and harbor high endemicity (Dimitrov, Nogués‐Bravo, & Scharff, 2012; Ricketts et al., 2005). They are also hotspots of extinction risk, due to the presence of threatened species with restricted distributions (Hoffmann et al., 2010; Ricketts et al., 2005). Montane specialists may also be threatened by climate change, which can trigger elevational range shifts (Stuhldreher & Fartmann, 2018). Where such shifts are constrained by topography, species may face habitat decline and local extinctions (Forero‐Medina, Joppa, & Pimm, 2011; Freeman, Scholer, Ruiz‐Gutierrez, & Fitzpatrick, 2018; Parmesan, 2006). Habitat losses often also cause habitat fragmentation, which has experimentally been shown to have negative effects on biodiversity and species persistence over and above the effects of habitat loss alone (Fahrig, 2003; Haddad et al., 2015). Declines in species abundances may occur after a significant time‐lag following an environmental perturbation, creating an extinction debt, and causing the effects of habitat disturbance to be underestimated (Haddad et al., 2015; Kuussaari et al., 2009; Tilman, May, Lehman, & Nowak, 1994). Globally, extinction debt averages over 20% and may affect as much as 75% of a local species assemblage (Haddad et al., 2015). The effects of climate change may interact with those of habitat loss and fragmentation (Fahrig, 2003), threatening montane habitats and the unique species assemblages they host.

The Western Ghats mountain range in southern India is a global biodiversity hotspot (Myers, Mittermeier, Mittermeier, Da Fonseca, & Kent, 2000) that includes locations of high extinction risk (Ricketts et al., 2005). The sky islands at the highest elevations of the Western Ghats host a naturally bi‐phasic mosaic of evergreen forest and grassland known as the *shola* ecosystem. Above 2,000 m, this ecosystem is dominated by montane grasslands (Thomas & Palmer, 2007; Das, Nagendra, Anand, & Bunyan, 2015), which harbor unique species assemblages (Biju, Garg, Gururaja, Shouche, & Walujkar, 2014; Sankaran, 2009). As with other tropical grasslands, these are poorly studied, despite the presence of several endemic species, and others of conservation concern (Bond & Parr, 2010).

This grassland biome faces severe anthropogenic threats. Although the forests are celebrated for their biodiversity, historically, the ecological role of the grasslands has not been recognized, and they have been intensively exploited for the establishment of commercial plantations (Joshi, Sankaran, & Ratnam, 2018). Many timber species thus introduced, including *Acacia mearnsii* (black wattle), *Eucalyptus* species, and *Pinus* species, have turned invasive (Thomas & Palmer, 2007; Arasumani et al., 2018; Joshi et al., 2018). Grassland loss to these species has been extensive and is variously estimated at 83% overall (Sukumar, Suresh, & Ramesh, 1995), 66% in the Palani hills region (Arasumani et al., 2018) and 38% overall (Arasumani et al., 2019), depending on the spatial scale and timeframe over which this change is measured. In addition to reducing habitat extent, the spread of exotic tree species has caused the grasslands, already a naturally patchy ecosystem (Robin, Gupta, Thatte, & Ramakrishnan, 2015), to become further fragmented. These changes threaten the wildlife of the habitat, including endemic species such as the Nilgiri tahr *Nilgiritragus hylocrius* (Rice, 1984) and nonendemics that are supported by the grasslands (Sankaran, 2009). Effective conservation of these habitat specialists therefore requires understanding factors determining the persistence of native grassland‐dependent species in the context of ongoing changes to the habitat.

The Nilgiri pipit, endemic to these montane grasslands, is an ideal case study to examine species persistence in this habitat. It is a locally common insectivore, resident in its breeding range, with no records of long‐distance movement (Vinod, 2007). Additionally, the effects of habitat characteristics on the distribution and abundance of the Nilgiri pipit are of wider interest in applied ecology: though features at both local and landscape scales have been shown to affect the presence and abundance of grassland birds (Jacoboski, Paulsen, & Hartz, 2017), their habitat requirements in montane habitats have received little attention. Changes to vegetation structure, including dominant grass height, in native grasslands has been found to favor habitat generalists over bird species that were dependent on specific grass types (Jacoboski et al., 2017). Microhabitat diversity within grasslands has been found to support a broader suite of grassland species (Dias, Bastazini, & Gianluca, 2014) and increased abundance of grassland‐dependent species (Azpiroz et al., 2012; Muchai, Lens, & Bennun, 2002). More generally, structural changes in vegetation can affect species even in areas where native vegetation cover is high (Fischer & Lindenmayer, 2007). Habitat heterogeneity in grasslands and montane habitats has generally received little attention (Tews et al., 2004). At the scale of a habitat fragment, area and isolation of grassland both have been found to affect grassland bird populations. Fragment area usually affects species' occurrence; however, this effect may disappear beyond a certain threshold, which may be species‐specific (Guttery et al., 2017). Local and landscape‐level factors can have additive effects on occupancy (Reidy, Thompson, Amundson, & O'Donnell, 2016). Features that create heterogeneity at small scales may be responsible for fragmentation at larger scales (Tews et al., 2004): Such features may therefore have complex impacts on occupancy and abundance, depending on the scale at which they are examined (Blevins & With, 2011).

Finally, the Nilgiri pipit is itself a species of conservation concern. It is classified as vulnerable by the IUCN, due to its small and fragmented range, which is declining in both extent and quality (BirdLife International, 2018). Recent surveys have failed to detect the species across a significant portion of its historical range (Robin, Vishnudas, & Ramakrishnan, 2014; Vinod, 2007), suggesting that the contemporary range of the species is much smaller than expected. In this context, examining the habitat factors allowing the species to persist assumes greater importance. In this study, we assessed factors driving patterns of distribution and abundance of the Nilgiri pipit across most of its known range. We used the single‐season occupancy model of Mackenzie et al. (2002) to understand the factors driving distribution and the *N*‐mixture model of Royle (2004) to understand grassland patch‐specific variation in abundance. Specifically, we explored the following, nonmutually exclusive hypotheses; that the Nilgiri pipit's presence and density would be positively affected by elevation; positively affected by microhabitat variation; positively affected by grassland patch size; negatively affected by exotic vegetation; and negatively affected by habitat isolation (Table 1).

**TABLE 1:** Description of variables used as predictors of Nilgiri pipit occupancy, abundance, and individual‐level detectability

Variable	Code	Definition	Data source	Expected effects on occupancy	Expected effects on abundance	Expected effects on detectability
Max. Elevation	MXELEV	The maximum elevation within a site	Remotely sensed ASTER GDEM data	++	++	*N*
Patch size	PCHSZ	Grassland patch size, log transformed	Remotely sensed Sentinel 2 data	+	+	*N*
Large grassland separation	LGSEP	Distance to nearest grassland > 1.5 km^2^, log transformed		+	+	*N*
Grassland within 500 m	GW500	Grassland area within 500 m of site		+	+	*N*
Wattle maturity	WTMAT	Categorical: None, Immature, or Mature *Acacia mearnsii*, judged by height and die‐off	Field observations	−	−	−
*Eucalyptus*	EUC	Presence or absence of *Eucalyptus* trees		−	−	−
Water	WAT	Presence or absence or running or flowing water		+	+	*N*
Burn	BU	Presence of an area burned between 1 and 12 months before the survey		+	+	+
Rhododendron	RHODO	Presence or absence of *Rhododendron*		+	+	+
Grass height	GH	Categorical: Short when predominantly < 15 cm, Tall when predominantly > 75 cm, Intermediate otherwise		*	*	−
Plantation extent	PLEXT	Proportion of 100 m × 100 m cells within a site occupied by wattle, *Eucalyptus*, or Pine	Google Earth imagery + Field observations	−	−	−
*Weather*	*WTHR*	*Categorical: Sunny, Overcast, or Foggy*	*Field observations*	*N*	*N*	‡
*Day*	*DAY*	*Number of days since the first survey*		*N*	*N*	+
*Time*	*TIME*	*Time difference from solar noon*		*N*	*N*	++

Note

Visit‐level variables are indicated in italics. Expected effects on species‐level detectability include expected effects on individual‐level detectability effects and expected effects on abundance.

*For grass height, occupancy and abundance were expected to be highest for the intermediate category. ^‡^Detectability was expected to be low in foggy weather, higher in sunny weather, and highest in overcast weather. All expectations were derived a priori from Vinod (2007; personal communication, 2018), Robin , Vishnudas, & Ramakrishna (2014) and preliminary field surveys.

Abbreviations: −, a negative effect; +, a positive expected effect; ++, a strongly positive expected effect; *N*, no expected effect.

## 2 MATERIALS AND METHODS

### 2.1 Study area

**FIGURE 1:**
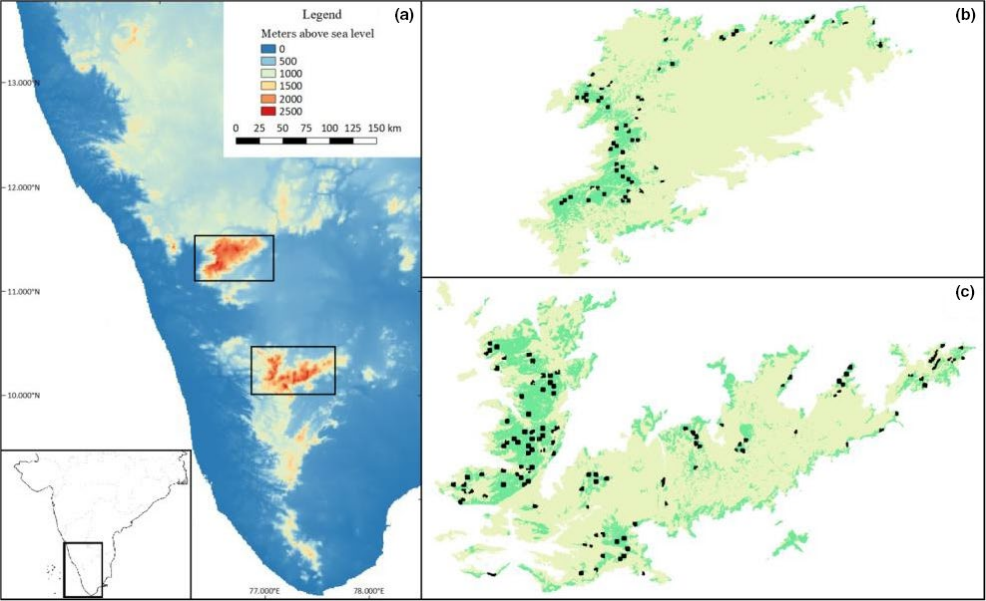
(a) Map depicting the sky islands of the Western Ghats, and their position in the Indian subcontinent (inset). (b, c) The grasslands (green) and sampling locations (black) in the Nilgiris (b) and the Anamalai‐Palani Hills (c): areas in yellow are above the 1,600 m contour

Robin , Vishnudas, & Ramakrishnan (2014) suggested that the Nilgiri pipit was restricted to areas above 1,900 m above sea level (a.s.l.). Conservatively, we limited our survey to grasslands above 1,600 m a.s.l. and also confined our study to areas in which verifiable contemporary records of the Nilgiri pipit exist. This region encompasses the two major high‐altitude plateaux of the Western Ghats; the Nilgiris and the Anamalai‐Palani Hills. Although the species has been reported elsewhere, photographic evidence or capture records for these locations do not exist and intensive surveys across the smaller northern and southern grasslands have failed to detect the species (Robin & Sukumar, 2002; Robin, Sukumar, & Thiollay, 2006; Sasikumar, Vishnudas, Raju, Vinayan, & Shebin, 2011) (Figure 1).

Within the area thus selected, we mapped the extent of montane grasslands using Sentinel‐2A imagery. We obtained imagery from the USGS Global Visualization Viewer (GloVis; https://glovis.usgs.gov/). Satellite imagery was obtained from the dry season (February 2017), when cloud cover was low. We removed atmospheric components such as dust particles, water vapor, and atmospheric temperatures in the satellite images by generating ground reflectance images using the Sen2Cor processor in SNAP v. 5.0.8 (ESA, 2017). We used a combination of supervised and unsupervised classification to map montane grasslands (see supplementary methods for further detail). The overall accuracy of this classification, determined using 100 ground‐truth GPS locations across the entire study area, was 96.5%, while the Kappa coefficient was 0.93 (following Congalton, 1991).

The final selected area represented 434.98 km^2^, or 85%, of the 511 km^2^ of grassland above 1,600 m in the Western Ghats. It consisted of 1,449 discrete grassland patches, varying in size from <1 ha to 120,000 ha. These extremes represented patches that were far too small to host independent Nilgiri pipits, to patches that could have had considerable variation in occupancy and density within them. We therefore treated the grassland patches in three ways, depending on their size. Grassland patches between 4 and 25 ha were designated as sample units, encompassing the range of our estimates for Nilgiri pipit home range (Vinod, 2007; personal communication, 2018). We laid a 500 m grid across all grassland patches larger than 25 ha, designated each grid cell a separate site, and removed all patches smaller than 4 ha. We placed patches between 1 and 4 ha into clusters, if each patch was within a maximum of 200 m away from at least one other (based on information about pipit movement; Vinod, personal communication, 2018), ensuring that slightly fragmented grasslands effectively larger than 4 ha were not discarded. We determined the total area of each cluster, and discarded clusters or individual patches totaling less than 4 ha as these were unlikely to support the species' occurrence. We randomly selected 202 sites from the remaining 2,378 potential sampling units and surveyed 170 of these (see Methods in Appendix S1 for more details of survey design and site selection).

### 2.2 Survey methods

To allow us to disentangle ecological processes shaping the presence and density of Nilgiri pipits from confounding processes related to observation that affected where pipits were more or less likely to be seen when present, we visited each site multiple times (maximum visits = 4:11 sites visited three times, one site twice, one site once). In order to equalize sample effort per unit area (and, therefore, detection probability per unit area) across patches of unequal size, the duration of each visit was set proportional to site area, with the time spent moving through each site and searching for the species. We expended 2 min of survey effort per hectare, which we determined to be optimal based on reconnaissance surveys. Thus, surveys were between 8 and 50 min long. Surveys were conducted on foot; Nilgiri pipit surveys were recorded based on visual and auditory detections strictly within the sampling site. Surveys attempted to cover the sampling site as completely as possible given the limitations of rugged terrain. Surveys were conducted between October 2017 and April 2018, during a single dry season in the study region.

Covariates were recorded at the site‐level and the visit‐level (covariates used in analyses listed in Table S1: see Appendix S1 for further details). Site‐level covariates were generated either from GIS data or field observations: Visit‐level covariates were recorded in the field. Of the 22 site‐level independent variables measured, six were eliminated based on collinearity with other independent variables, and five were eliminated because there was insufficient variation in them across our sites. Covariates displaying moderate collinearity were not included within the same model. Of the 11 remaining covariates, two were expected to affect abundance but not occupancy, and some were used only as detection covariates in modeling pipit occupancy.

Covariates included the presence or absence of two types of exotic vegetation, black wattle (*Acacia mearnsii*), and *Eucalyptus*, both known as invasive taxa contributing to grassland loss; and one native taxon, *Rhododendron*, which may be expected to influence local habitat heterogeneity. The extent to which all exotic trees were present within the site was also recorded. The presence or absence of recent burns was recorded, as accidental and controlled burns are both regular features of this landscape, and may be expected to affect habitat suitability. Distance to large grasslands was included to examine whether dispersal from large patches was shaping occupancy in small patches; grassland extent within a buffer zone was assessed to determine whether pipits required functionally larger patches than their observed home range size. The size of this buffer was chosen to be 500 m, based on Vinod's observations (2007) of Nilgiri pipit movement. Microhabitat type within grasslands had previously been observed to affect pipit presence (Vinod, 2007, personal communication, 2018); grass height and the presence of water sources were included as covariates to assess this effect.

Visit‐level covariates included the weather, date, time of day, and observer identity. Weather was observed to have effects on detection during preliminary surveys. The effect of observer identity was assessed in preliminary analysis, but was not used in subsequent modeling, as no substantial variation was seen. The number of days since the first survey was recorded as a proxy for season. Time of day was transformed into time away from solar noon, to account from a known bimodal pattern in the diurnal activity of the Nilgiri pipit (Vinod, 2007, personal communication, 2018).

### 2.3 Statistical analysis

We first examined our covariates and eliminated some based on extreme collinearity between covariates or insufficient variation among our sites. Our expected effects on (i) individual detection, (ii) occupancy, (iii) species‐level detection, and (iv) density (Table 1) were based on previous literature (Robin, Vishnudas, & Ramakrishnan, 2014; Vinod, 2007), our collective prior knowledge of the species' biology, and preliminary surveys. Based on our understanding of Nilgiri pipit biology, we constructed plausible combinations of covariates that we expected to influence (i)–(iv) above as a first step in constructing models of variation in Nilgiri pipit occupancy or abundance. Covariates that had substantial, but not extreme, collinearity (Pearson's |*r*| > .7; Dormann et al., 2013) were retained in the model set but never included in the same model.

In investigating variation in either pipit occurrence (using occupancy modeling) or pipit abundance (using *N*‐mixture modeling), we were faced with the intractable problem of having to model all plausible combinations of covariates for the observation process (species‐level detection in occupancy modeling; individual‐level detection in *N*‐mixture modeling) with all combinations of the covariates for occupancy or abundance, respectively. We therefore chose to use a two‐step modeling procedure, where we used a fairly general covariate structure to describe variation in the occupancy and abundance while examining various covariate combinations for the observation processes. The most supported covariate combinations for the observation processes were then used with various covariate combinations for occupancy and for abundance, to determine the final best‐supported model (see Doherty, White, and Burnham (2012) for a discussion of such two‐step modeling procedures). We recognize that this precludes some analytical options, such as the use of summed Akaike weights to assess covariate importance, as the model set is inherently unbalanced. Analyses were carried out using the package “unmarked” (Fiske & Chandler, 2011) in the statistical software R (R Core team, 2018).

#### 2.3.1 Modeling Nilgiri pipit occupancy

Counts were reduced to detections and nondetections for the occupancy analysis. We used a combination of three covariates to model variation in probability of occupancy while assessing support for different covariate combinations for modeling variation in probability of detection. We modeled additive effects of combinations of covariates, except in the case of time and weather, which we expected to interact in determining detectability. By setting our survey duration per site proportional to the area of that site, we ensured that species detection was equal across sites of variable size, thus dispensing with the need to include site area as a covariate for detectability. Model selection was carried out based on Akaike's information criterion (AIC). Conservatively sampling to the bottom of the Nilgiri pipit's known elevational range, as we did, created the possibility that our analysis was predisposed to detect a strong effect of elevation. We therefore repeated our analysis on the subset of sites for which the maximum elevation was greater than 1,800 m a.s.l. (*N* = 151).

We tested 127 detection covariate combinations using three covariates to model occupancy and used the best‐fitting combination to fit 56 occupancy covariate combinations. The best‐fitting occupancy covariate combinations (AIC weight > 0.02) are in Table 2. Adequate model fit (*p* = .8392) was indicated by the chi‐square goodness‐of‐fit test from 5,000 parametric bootstrap simulations on the most general model (MacKenzie & Bailey, 2004), for the subset of sites visited four times (*N* = 156); we excluded sites visited fewer than four times from our assessment of goodness‐of‐fit, as sample sizes were too low to treat these as separate cohorts. Since no single model received overwhelming support in the second step, model‐averaged predictions were used to derive response curves for each covariate (Burnham & Anderson, 2002). Since the final set of sites visited was nonrandom due to attrition of sites (see Appendix S1 for details), we did not attempt to estimate overall occupancy (proportion of area occupied) of Nilgiri pipits across the survey landscape.

**TABLE 2:** Estimated *β* coefficients for each predictor of occupancy from models with AIC weight ≥ 0.02

Occupancy structure	β MXELEV	β PCHSZ	β LGSEP	β WTMAT		β EUC	β PLEXT	β RHODO	β GH		AIC	∆AIC	AIC Weight	Cum. AIC weight
				Imm.	Mat.				Int.	Tall				
MXELEV + LGSEP	9.01 ± 2.68		−0.570 ± 0.266								537	0	0.173	0.173
MXELEV + PCHSZ	8.86 ± 2.43	0.573 ± 0.251									537	0.04	0.170	0.343
MXELEV + LGSEP + PLEXT	9.55 ± 2.62		−0.466 ± 0.277				−1.37 ± 1.33				538	1.11	0.0992	0.442
MXELEV + PCHSZ + PLEXT	9.39 ± 2.56	0.464 ± 0.299					−0.939 ± 1.42				538	1.61	0.0772	0.520
MXELEV + PLEXT	10.8 ± 2.57						−2.09 ± 1.21				539	1.79	0.0708	0.590
MXELEV	10.7 ± 2.83										539	2.05	0.0623	0.653
MXELEV + WATMAT	13.0 ± 3.45			−2.45 ± 1.58	−2.15 ± 1.14						539	2.05	0.0623	0.715
MXELEV + PLEXT + GH	9.34 ± 2.23						−2.31 ± 1.07		1.78 ± 1.09	−0.44 ± 1.48	539	2.45	0.0506	0.766
MXELEV + PCHSZ + RHODO + GH	7.75 ± 2.69	0.548 ± 0.222						0.123 ± 0.888	1.61 ± 0.981	−0.15 ± 1.57	540	3.51	0.0300	0.795
MXELEV + PLEXT + RHODO	11.4 ± 3.38						−2.21 ± 1.32	−0.348 ± 1.02			540	3.67	0.0277	0.823
MXELEV + RHODO	11.6 ± 3.99							−0.437 ± 1.13			541	3.88	0.0249	0.848
MXELEV + EUC	10.4 ± 2.87					−0.314 ± 0.942					541	3.94	0.0241	0.872
MXELEV + LGSEP + RHODO + GH	7.71 ± 2.76		−0.533 ± 0.239					0.0134 ± 0.947	1.49 ± 1.11	−1.09 ± 1.55	541	4.04	0.0230	0.895
MXELEV + LGSEP + PLEXT + RHODO + GH	8.68 ± 3.17		−0.385 ± 0.256				−0.165 ± 1.22	−0.0461 ± 1.01	1.53 ± 1.04	−0.696 ± 1.44	541	4.26	0.0205	0.915

Note

In each model presented below, detectability was modeled as a function of (Weather + Day + Grass height + Wattle maturity + Eucalyptus + Rhododendron + Water + Burn + Grassland within 500 m). Variable abbreviations are provided in Table 1.

#### 2.3.2 Modeling Nilgiri pipit abundance

We used model‐averaged occupancy predictions to estimate occupancy for each site. To avoid over‐dispersion in site‐specific abundance due to zero‐inflation (Joseph, Elkin, Martin, & Possingham, 2009), only 112 sites with a predicted occupancy above 0.4, based on a clear threshold in a plot of estimated occupancy, were used for analysis using *N*‐mixture models (Royle, 2004). A total of 22 detection covariate combinations were tested with a global abundance covariate combination. We used the best‐fitting covariate combination for detection to fit 91 covariate combinations for abundance: Six had an AIC weight of >0.02 (Table 3). Seven of the 11 independent variables appeared in covariate combinations with substantial support. The area of a sampling site was expected to be proportional to the number of individuals observed in it; we therefore used log(site area) as an offset in all *N*‐mixture models to control for site area, and so estimates of site‐specific abundance λ may be interpreted as expected Nilgiri pipit density per hectare. Despite excluding sites with a low probability of occurrence, the chi‐square goodness‐of‐fit test based on 5,000 parametric bootstrap simulations implemented in the R package “AICcmodavg” (Mazerolle, 2016) indicated moderate over‐dispersion of latent abundances relative to the model ($\hat{c}$ = 1.93). Estimated $\hat{c}$ was therefore used to derive QAIC values for model selection and to adjust estimated variances.

**TABLE 3:** Estimated *β* coefficients for each predictor of abundance from models with QAIC weight ≥ 0.02

Abundance structure	β MXELEV	β WTMAT		β EUC	β WAT	β RHODO	β GH		β BU	QAIC	∆QAIC	QAICWeight	Cum. QAIC weight
		Imm.	Mat.				Int.	Tall					
MXELEV + WTMAT + EUC + WAT + GH + BU	1.424 ± 0.370	0.313 ± 0.189	−0.434 ± 0.247	−0.377 ± 0.168	0.504 ± 0.106		0.398 ± 0.193	−1.663 ± 1.010	0.231 ± 0.122	1,018.63	0	0.298	0.298
MXELEV + WTMAT + EUC + WAT + RHODO + GH + BU	1.303 ± 0.383	0.310 ± 0.188	−0.390 ± 0.247	−0.365 ± 0.168	0.464 ± 0.110	0.192 ± 0.147	0.371 ± 0.195	−1.661 ± 1.007	0.192 ± 0.125	1,018.92	0.281	0.259	0.557
MXELEV + WTMAT + EUC + WAT + RHODO + GH	1.310 ± 0.386	0.356 ± 0.187	−0.332 ± 0.246	−0.367 ± 0.169	0.474 ± 0.110	0.241 ± 0.143	0.303 ± 0.191	−1.713 ± 1.006		1,019.26	0.630	0.218	0.775
MXELEV + WTMAT + WAT + RHODO + GH + BU	1.24 ± 0.382	0.374 ± 0.184	−0.339 ± 0.247		0.471 ± 0.111	0.211 ± 0.148	0.410 ± 0.197	−1.725 ± 1.007	0.195 ± 0.126	1,021.68	3.046	0.065	0.840
MXELEV + WTMAT + WAT + GH + BU	1.380 ± 0.369	0.381 ± 0.185	−0.377 ± 0.247		0.511 ± 0.107		0.436 ± 0.196	−1.750 ± 1.018	0.234 ± 0.123	1,021.72	3.083	0.064	0.904
MXELEV + WTMAT + WAT + RHODO + GH	1.246 ± 0.385	0.412 ± 0.184	−0.287 ± 0.246		0.481 ± 0.111	0.258 ± 0.144	0.344 ± 0.193	−1.744 ± 0.988		1,022.07	3.434	0.054	0.957

Note

In each model presented below, detectability was modeled as a function of (Weather + Day +Plantation cover). Variable abbreviations are provided in Table 1.

## 3 RESULTS

### 3.1 Nilgiri pipit occupancy

**FIGURE 2:**
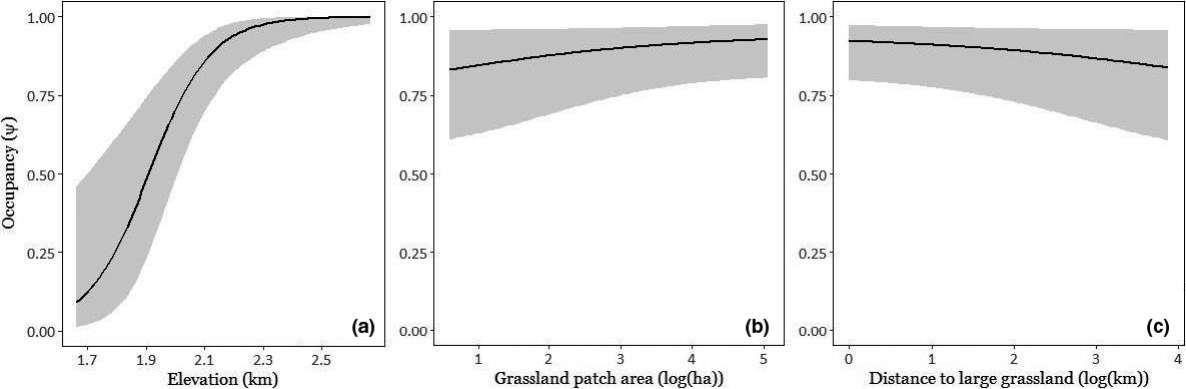
(a–c) Model‐averaged predicted occupancy of the Nilgiri pipit in response to the three covariates with the largest effects; maximum elevation within a site (a), log (grassland patch area) (b), and log (distance to the nearest grassland larger than 1.5 km^2^) (c). All other variables are set to mean values. Predicted probability of occupancy is plotted over the observed range of values of each predictor. Bands represent 95% confidence intervals

We detected the Nilgiri pipit in 109 of 170 sites (naïve occupancy = 0.641). The best‐fitting model for detectability, was “Weather + Day + Grass height + Wattle maturity + Eucalyptus + Rhododendron + Water + Burn + Grassland within 500 m.” Occupancy covariate combinations fit using this covariate combination for detection that had an AIC weight ≥0.02 are listed in Table 2. Model‐averaged results show that maximum site elevation had a strong positive effect on occupancy, while patch isolation and patch area (fit only in separate models, as they were strongly correlated) had moderate negative and positive correlations with occupancy, respectively (Figure 2). All 14 best‐fitting models included maximum site elevation as a covariate, while the top four included either patch isolation or grassland patch area. All but one (quantity of grassland within 500 m of the site) of the putative occupancy predictors appeared in one or more model with substantial support. Models comprising combinations of isolation, area, and elevation had comparable AIC weights to those including additional covariates: However, each of these other covariates had extremely small effects on occupancy. When the analysis was repeated on sites with maximum elevation greater than 1,800 m a.s.l., the identity of most models with high support did not change, and AIC weights were comparable (Table S2). Model‐averaged response curves showed relationships between habitat covariates and species presence indistinguishable from those found with the full data set (Figure S3).

### 3.2 Nilgiri pipit abundance

**FIGURE 3:**
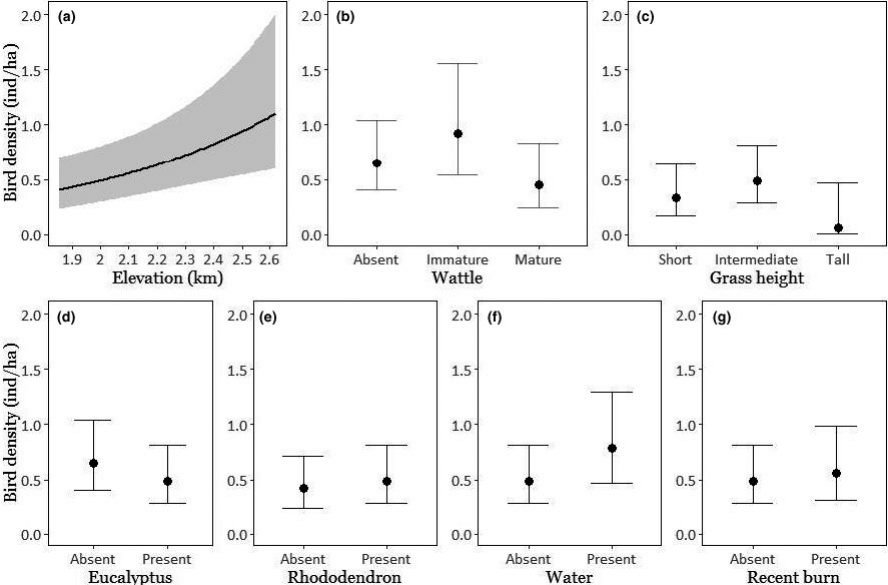
(a–g) Model‐averaged predicted density (per hectare) of the Nilgiri pipit in response to all covariates appearing in models with AIC weight > 0.01; maximum elevation within a site (a), maturity of wattle (b), grass height (c), presence of *Eucalyptus* (d), presence of *Rhododendron* (d), presence of water (f), and presence of a recently burned area (g). All other variables are set to mean or model values for continuous and categorical covariates, respectively. Predicted density is plotted over the observed range of values of each predictor. Band and error bars represent 95% confidence intervals

In the 144 sites with *ψ* > 0.4, we detected 0 to 14 individuals (mean = 3.76). The best‐supported covariate combination for detectability was “Weather + Day + Plantation cover.” Covariate combinations fit to density data using this detection covariate combination that had an AIC weight ≥ 0.02 are listed in Table 3. Model‐averaged results showed that elevation had a strongly positive effect on bird density: the predicted density at the maximum sampled elevation was more than twice that at the lowest elevation. The best‐fitting six models all included elevation, grass height, wattle maturity, and water, while *Rhododendron*, *Eucalyptus*, and recent burns appeared in three of the four best‐fitting models. *Eucalyptus* negatively affected density, while the presence of water positively affected density. The presence of both *Rhododendron* and burned area had marginally positive effects on density. Grassland patch size and isolation, and plantation extent, were not part of models that had any substantial support (Figure 3).

## 4 DISCUSSION

We found that maximum site elevation, grassland patch size, and distance to the nearest large grassland were the only covariates that had substantial effects on pipit occupancy. In contrast, abundance was shaped by maximum site elevation in combination with many site‐level habitat characteristics, each of which had a substantial effect on predicted abundance.

Nilgiri pipit occupancy and abundance both have strong relationships with elevation. Only sites above 1,800 m have high probabilities of occupancy. As there are no other montane grassland specialists in the Western Ghats (Rasmussen & Anderton, 2012), the Nilgiri pipit is likely to be the most elevationally restricted bird species in the Indian subcontinent south of the Himalaya. The only detection of Nilgiri pipits below 1,700 m was at the southwestern extremity of the Anamalai plateau, with anomalously high exposure to the southwest monsoon and a local climate consistent with higher areas elsewhere. This dependence of occurrence and abundance on elevation are similar to the distribution patterns of other montane flora, including *Rhododendron* (Giriraj et al., 2008), and fauna (Mizel, Schmidt, Mcintyre, & Roland, 2016; Yandow, Chalfoun, & Doak, 2015), including habitat‐specialist birds (Watson, 2003) like the *Sholicola* (Robin & Sukumar, 2002).

**FIGURE 4:**
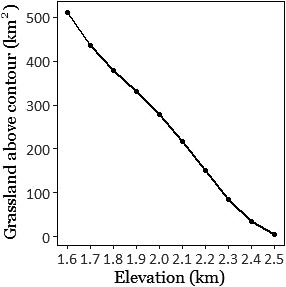
Total available grassland above successive 100 m contours in the Western Ghats. Available grassland declines rapidly with elevation, implying that the range of the Nilgiri pipit would decline rapidly if it is forced upward by climate change

This species–habitat relationship suggests that the Nilgiri pipit is likely to be extremely vulnerable to climate change. Vulnerability to climate change may be strongly influenced by species traits (MacLean & Beissinger, 2017; Pacifici et al., 2017). Additionally, our study is limited by our use of elevation as a proxy for bioclimatic conditions that directly affect the species; we emphasize that detailed investigation of the effects of these variables is necessary to better understand the ecology of the Nilgiri pipit and other montane grassland specialists. Nonetheless, we believe that the strength of the relationships between species presence and elevation, and abundance and elevation, clearly suggest that the species is highly dependent on extremely specific habitat conditions, which climate change is likely to disrupt. In particular, any upward shift in the species' elevational range is likely to lead to substantial range contraction (Parmesan, 2006; Sekercioglu, Schneider, Fay, & Loarie, 2008), given the distribution of area with respect to elevation in the two plateaux (Figure 4). Although such range shifts vary greatly in magnitude based on species ecology, the likelihood of such a shift is strengthened in this case by the nature of the montane grassland ecosystem. Montane grassland becomes the dominant biome only above 2,000 m (Das et al., 2015) and is maintained by temperature‐mediated frost regimes that are likely to be shifted by anthropogenic climate warming (Joshi, Ratnam, & Sankaran, 2019).

Any loss of habitat for range‐restricted species such as the Nilgiri pipit can be severely detrimental. Our sampling did not include patches of dense, mature, plantations of invasive woody species, as these habitats are not viable for grassland specialists. Within the sampled grasslands, the presence of these invasives did not affect the occupancy of the Nilgiri pipit, but negatively affected abundance. The exception was the patch condition “immature wattle,” which had a higher predicted abundance than either “mature wattle” or “no wattle.” We suggest two possible explanations for this anomaly. First, areas with immature wattle but without mature wattle largely (19 of 28 sites) occur within the two largest grassland patches, Eravikulam and Mukurthi National Parks, which have high Nilgiri pipit densities, and where management practices include removal of mature wattle. Second, immature wattle may temporarily contribute to habitat heterogeneity (a factor that increases abundance, as discussed below) without substantially degrading habitat quality. However, due to wattle's rapid growth, this effect is likely to be transient.

Our findings suggest that while Nilgiri pipit presence may be constrained by habitat availability, its abundance is shaped by local habitat quality. It is probable that low‐density populations in areas affected by invasive species are nonviable. Woody monocultures are driving declines in abundance: Such declines may cause functional extinction even in areas where the species is present (Dirzo et al., 2014). Furthermore, woody invasives are replacing grassland over longer timescales (Arasumani et al., 2018) and thereby are likely to also constrain occupancy. Several studies have found detrimental impacts of spreading woody exotic species on grassland avifauna, including in the Brazilian Pampas (Azpiroz et al., 2012; Jacoboski, Paulsen, & Hartz, 2017) and South African highland grasslands (Allan, Harrison, Navarro, Van Wilgen, & Thompson, 1997), suggesting that such a spread is a widespread phenomenon globally, requiring broader attention.

In grasslands that are remnants of century‐old habitat loss in the eastern Nilgiris (Joshi et al., 2018), we found a complete absence of Nilgiri pipits (Figure S1), but grassland remnants in the Palani Hills, invaded and fragmented severely since 1973 (Arasumani et al., 2018) supported low‐density populations. We conclude that grasslands in the eastern Nilgiris have experienced local extinctions, as historical records clearly indicate the species' presence in that region (Robin, Vishnudas, & Ramakrishnan, 2014). Furthermore, high‐altitude habitat specialists are often strongly affected by patch area (Watson, 2003), but Nilgiri pipit presence showed only a weak correlation with patch size and isolation, which is unexpected for a poor disperser (Rosenzweig, 1995), such as the Nilgiri pipit (Vinod, personal communication, 2018). We note that this species–habitat relationship is specific to the landscape under consideration; in this case, grassland patches deemed large enough to encompass the home range of an individual Nilgiri pipit. Exploring whether and how the species uses smaller patches may change the strength of the relationship with patch size and isolation. These findings may represent a substantial extinct debt in grassland recently affected by invasive species in the Palani hills and the southern Anamalais. The effects of local extinctions on population structure and viability is likely to be stronger in non‐avian grassland endemics, since birds have greater dispersal abilities (Watson, 2003), particularly in long‐lived species that are likely to have greater extinction debt (Krauss et al., 2010).

The abundance of the Nilgiri pipit showed a strong positive correlation with intermediate or mixed grass height: such a preference for specific grass height has also been documented for a suite of avian species in the Brazilian pampas (Jacoboski, Paulsen, & Hartz, 2017). We found moderate positive correlations with other variables contributing to local habitat heterogeneity and vegetation structure, a correlation supported by the behavioral observations of Vinod (2007), who found that Nilgiri pipits preferred marshy habitat with tall grass for nesting, and more open habitat for feeding. We note that although grass height may be a labile variable at the scale of a grassland patch, the categories we used reflect variation in the nature of available grassland at the landscape scale. The response of grassland birds to habitat heterogeneity is complex, with both positive and negative relationships documented (Pavlacky, Possingham, & Goldizen, 2015; Wiens & Rotenberry, 1981). While further investigation of the functional effects of structural complexity in the montane grasslands of the Western Ghats is merited, our findings show a broad dependence on natural habitat heterogeneity within this habitat.

Our findings have several implications for land management. The contiguous grassland within Eravikulam and Grasshills National Parks is the only remaining patch with high pipit abundance without large areas lost to invasive species: Its continued protection is therefore of critical importance. The grassland in and around Mukurthi National Park, containing the only other high‐density population, is rapidly shrinking due to invasion (Arasumani et al., 2019), largely by black wattle, and requires urgent management attention. Conversely, many grasslands in which we suspect high potential for extinction are outside protected areas and have already been severely fragmented. These require a different management strategy targeting connectivity and restoration: such strategies have already been explored at local scales (Mudappa, D., & Shankar Raman, T. R., personal communication, 2018; Stewart, R., personal communication, 2018).

We found that patch‐level habitat quality had a strong effect on abundance: Such a pattern has also been found in other highland avifauna (Allan, Harrison, Navarro, Van Wilgen, & Thompson,   1997; Watson, 2003). Thus, conservation efforts must focus on maintaining habitat quality over and above simply preserving grassland. Frequent fire is thought to positively affect grassland avian species richness (Pons, Lambert, Rigolot, & Prodon, 2003). We did not find a strong relationship between recent burns and Nilgiri pipit density: Further and more systematic study is required to draw any conclusions about the role of fire in management in this landscape. At a local scale, the presence of woody invasives reduces Nilgiri pipit abundance, while at the landscape scale, the spread of the same woody invasives shapes grassland patch size and isolation (Arasumani et al., 2019), which drive Nilgiri pipit occupancy. We emphasize that our study was limited to the remnant grasslands, and our findings therefore greatly underestimate the detrimental effects of invasive vegetation.ompletely wooded habitats that were previously grasslands were not sampled, since these do not currently have any Nilgiri pipits. Controlling the spread of invasive tree species into high elevation montane grasslands is a matter of urgency for conservation.

## 5 CONCLUSIONS

We demonstrate that elevation shapes both occupancy and abundance of this montane specialist. Furthermore, our study demonstrates that woody invasives are constraining occupancy via rapid grassland loss at the landscape level, while also degrading habitat quality at the local level. Our research also indicates local extinctions in large parts of the species’ range. This study underscores the urgent need for conservation actions targeted at the poorly known montane grasslands and the specialist species dependent upon it.

## CONFLICT OF INTEREST

The authors declare that they have no conflict of interest.

## AUTHOR CONTRIBUTION

**Abhimanyu Lele:** Conceptualization (equal); Data curation (equal); Formal analysis (lead); Funding acquisition (equal); Investigation (lead); Methodology (supporting); Project administration (equal); Resources (equal); Software (lead); Visualization (lead); Writing‐original draft (lead); Writing‐review & editing (equal). **M. Arasumani:** Data curation (equal); Formal analysis (supporting); Investigation (supporting); Software (supporting); Visualization (supporting); Writing‐review & editing (equal). **C. K. Vishnudas:** Conceptualization (equal); Funding acquisition (equal); Investigation (supporting); Methodology (supporting); Validation (equal); Writing‐review & editing (equal). **Viral Joshi:** Investigation (supporting); Validation (equal); Writing‐review & editing (equal). **Devcharan Jathanna:** Conceptualization (equal); Formal analysis (supporting); Methodology (lead); Software (supporting); Writing‐review & editing (equal). **V. V. Robin:** Conceptualization (equal); Formal analysis (supporting); Funding acquisition (equal); Methodology (supporting); Project administration (equal); Resources (equal); Supervision (lead); Validation (equal); Writing‐review & editing (equal).

## Supporting information

Appendix S1

## Data Availability

The data that support the findings of this study are openly available on the data repository *Dryad* at https://doi.org/10.5061/dryad.ksn02v70t
