# Supplementary material for: Elevation and landscape change drive the distribution of a montane, endemic grassland bird
Source: Ecol Evol. 2020 Jul 6;10(14):7755–67. doi: 10.1002/ece3.6500 (PMC7391316; doi:10.1002/ece3.6500)
Supplement: Supplementary file 1 — Appendix S1 [file ECE3-10-7755-s001.docx]

**10.0 Supplementary methods**

**10.1 Administrative regions examined in the study**

Our study area fell within the following administrative regions. In the Nilgiris, our surveys were conducted within Mukurthi National Park, Nilgiri South Forest Division, and Nilgiri North Forest Division, all units of the Tamil Nadu Forest Department. In the Anamalai- Palani Hills region, our surveys were carried out in Grasshills National Park, Kodaikanal Wildlife Sanctuary, and Dindigul Forest Division (under the Tamil Nadu Forest Department), and Eravikulam National Park and Munnar Wildlife Division under the Kerala Wildlife Department. A few survey locations were located within private land, in which case permission was obtained from the owners to conduct surveys therein.

**10.2 Mapping grasslands using remote sensing**

We used Sentinel – 2A data for montane grassland mapping. Satellite images were downloaded from USGS Global Visualization Viewer (GloVis; https://glovis.usgs.gov/): the images acquired were from February and March 2017. We selected images with less than 1% cloud cover from GloVis and acquired them for further processing. Nine Sentinel 2A scenes covered our study area. We mosaicked all the satellite images using SNAP v. 5.0.8. and performed a geometric correction to improve geolocation of each pixel: the root mean square error was less than half a pixel. We corrected for atmospheric distortion by removing atmospheric components (e.g. water vapour column, dust particles and aerosol) using the Sen2cor processor in SNAP v5.0.8. We used four bands (Band 2 – 192.4 nm, Band 39 - 559.8, Band 4 - 664.6 and Band 8 – 832.8) with 10m spatial resolution for image classification. Our study area was divided into 5 km^2^ grid cells for training sample collection and ground truth verification. We collected 223 training samples between April 2017 and September 2017 across the study area for image classification. We interpreted the nine Sentinel 2A scenes using the hybrid classification approach, which combines digital supervised classification, unsupervised classification, and topography (Arasumani et al. 2019). The accuracy of the classified map was calculated using ground truth GPS points. We created 100 random points on the map and visited each of them to evaluate the accuracy of our classification. Overall accuracy was found to be 96.5%, and the kappa coefficient was 0.93 (following Congalton, 1991).

**10.2 Sampling sites and site selection**

Of the 3012 grassland sampling units which remained after patches deemed too small to support pipits had been removed from our sampling frame (*sensu* Williams, Nichols, & Conroy, (2002)), 300 were randomly selected to be surveyed for detection/non-detection of Nilgiri pipits. Of these 300, areas containing 69 sites were removed because they were determined by the relevant government agency to be unsafe due to armed militant activity, or for other reasons of accessibility. The remaining study landscape included 234 sites. Of these 234, 8 sites were removed due to misclassification: they were found to be *Eucalyptus* plantation or scrub forest, and in 3 cases, to be under water, because they flooded between when classification was carried out and when the surveys were conducted. A further 21 sites could not be sampled as they were directly adjacent to other (sampled) sites, and Nilgiri pipit presence in them could not be assumed to be independent of all other sites. Thus, the total area of grassland sampled was 434.98km^2^, which contained 202 sites that fit our criteria for sampling (accurate habitat classification, and independence from other sites. Of these 202, 170, or 84%, were sampled: 32 sites were found to be physically inaccessible due to the topography of the landscape or the ownership of the land.

**10.3 Covariates**

Landscape-level covariates were generated from GIS data. Site-level covariates were generated either from GIS data or field observations. Visit-level covariates were measured in the field. A full list of covariates is provided below. Variables used in the final analyses are in **boldface** font. Of the 22 site-level independent variables, we eliminated some variables based on collinearity. Five variables were eliminated because there was insufficient variation in these across our sites. Any other covariates displaying moderate collinearity were not included within the same model. Of the 11 remaining covariates, two were expected to affect abundance but not occupancy, and some were used only as detection covariates in modelling pipit occupancy.

**Supplementary Table 1: All covariates**

| **Variable** | **Definition** | **Data source** |
| --- | --- | --- |
| **Max. Elevation** | **The maximum elevation within a site, based on 30m resolution imagery** | Remotely-sensed ASTER GDEM data |
| Mean Elevation | The mean elevation within a site**, based on 30m resolution imagery** |  |
| Min. Elevation | The minimum elevation within a site**, based on 30m resolution imagery** |  |
| Slope | The mean slope within a site**, based on 30m resolution; calculated by processing Aster GDEM images in ArcGIS 10.5** |  |
| **Patch size** | **Grassland patch size, log transformed** | Remotely-sensed Sentinel 3 data |
| **Large grassland separation** | **Distance to nearest grassland >1.5km^2^, log transformed** |  |
| **Grassland within 500m** | **Grassland area within 500m of site** |  |
| Grassland within 1000m | Grassland area within 1000m of site |  |
| **Plantation extent** | **Proportion of 100m × 100m cells within a site occupied by wattle, *Eucalyptus*, or Pine** | Google Earth imagery + Field observations |
| **Wattle maturity** | **Categorical: None, Immature, or Mature *Acacia mearnsii*, judged by height and the presence of die-offs.** | Field observations |
| **Eucalyptus** | **Presence or absence of trees in the genus *Eucalyptus*** |  |
| Pine | Presence or absence of trees in the genus *Pinus* |  |
|  |  |  |
| **Water** | **Presence or absence or running or flowing water** |  |
| Marsh | Presence or absence of marshy area |  |
| **Burn** | **Presence of an area burned between 1 and 12 months before the survey** |  |
| **Rhododendron** | **Presence or absence of genus *Rhododendron*** |  |
| Gorse | Presence or absence of genus *Ulex* |  |
| Scotch Broom | Presence or absence of *Cytisus scoparius* |  |
| Lantana | Presence or absence of *Lantana camara* |  |
| Eupatorium | Presence or absence of *Eupatorium adenophorum* |  |
| Other native shrubs | Presence or absence of other native shrubs, primarily *Strobilanthes* |  |
| **Grass height** | **Categorical: Short when predominantly <15cm, Tall when predominantly >75cm, Intermediate otherwise** |  |
| ***Weather*** | ***Categorical: Sunny, Overcast, or Foggy*** | *Field observations* |
| ***Day*** | ***Number of days since the first survey*** |  |
| ***Time*** | ***Time difference from solar noon; calculated using site longitude.*** |  |
| *Observer* | *Identity of the most experienced observer on a survey* |  |

Covariates included the presence or absence of the following categories of vegetation; black wattle (*Acacia mearnsii*), *Eucalyptus*, pine (genus *Pinus*), *Lantana camara*, *Eupatorium adenophorum* (also known as *Ageratina adenophora*), *Rhododendron*, and other native shrubs, including *Strobilanthes spp.* The first five of these vegetation types are common invasive exotics in the high-altitude grassland landscape; the other two covariates represent the other significant types of vegetation found within the grassland, which may be expected to influence local habitat heterogeneity within the grasslands. Data for *A. mearnsii* were divided into the categories “Mature” and “Immature”, as each category was expected a priori to affect pipits differently and these also seemed to accurately reflect variation on the ground; several sites had experienced recent invasion from *A. mearnsii* and had not yet been substantially altered by this invasion. Furthermore, black wattle trees experience die-offs beyond a certain size, which are easily identifiable in the field, providing a good proxy for the maturity and hence density of a stand of wattle. The presence or absence of water sources was recorded, as previous observations suggested pipits breed near water sources (Vinod 2007, personal communication, 2018). The presence or absence of recent burns was included as a covariate due to the presence of accidental burns in many areas, and the use of controlled burning as a management technique in Eravikulam National Park, which constitutes the largest single management unit within our study area.

The threshold of 1.5km^2^ for defining a “large grassland” was based on observations which found that virtually all pipit populations which appeared large and healthy, and where pipits were regularly detected in large numbers, were in patches larger than 1.5km^2^.

The predominant height of the grass within a site was a categorical variable with three levels. Grass was characterised as short when it was shorter than 15cm, tall when it was taller than 75cm, and intermediate in all other cases. This categorization was based on observations of different microhabitats. Cliffs, heavily grazed areas, or recently burned areas had extremely short grass; marshy areas and scrub habitat found up to approximately 1800m had very tall grass; the ‘intermediate’ category typically included native montane grasslands and more mixed grassland.

Visit-level covariates included the weather, date, time of day, and identity of the most experienced observer. Weather was recorded as a categorical variable with three levels; sunny, overcast, and foggy, as these types of weather had clear effects on detection. Data were not collected during rain. Observer identity was recorded to assess potential differences in detection efficacy between observers. The number of days since the first survey was recorded as a proxy for season, as seasonal variation in the Nilgiri pipit’s behaviour was known (Vinod 2007) and needed to be controlled for. Similarly, the diurnal activity of the Nilgiri pipit was known to have a bimodal pattern with peaks soon after sunrise and shortly before sunset: thus, we transformed time of day to time away from solar noon for analysis.

Gorse, Scotch Broom, Pine, and *Lantana* were eliminated as variables because they were found to be present in <10% of the sites surveyed. *Eupatorium* and the “Other native shrubs” variable were eliminated because they were found in >95% of the sites surveyed.

**10.4 Spatial variation in Nilgiri pipit detection**


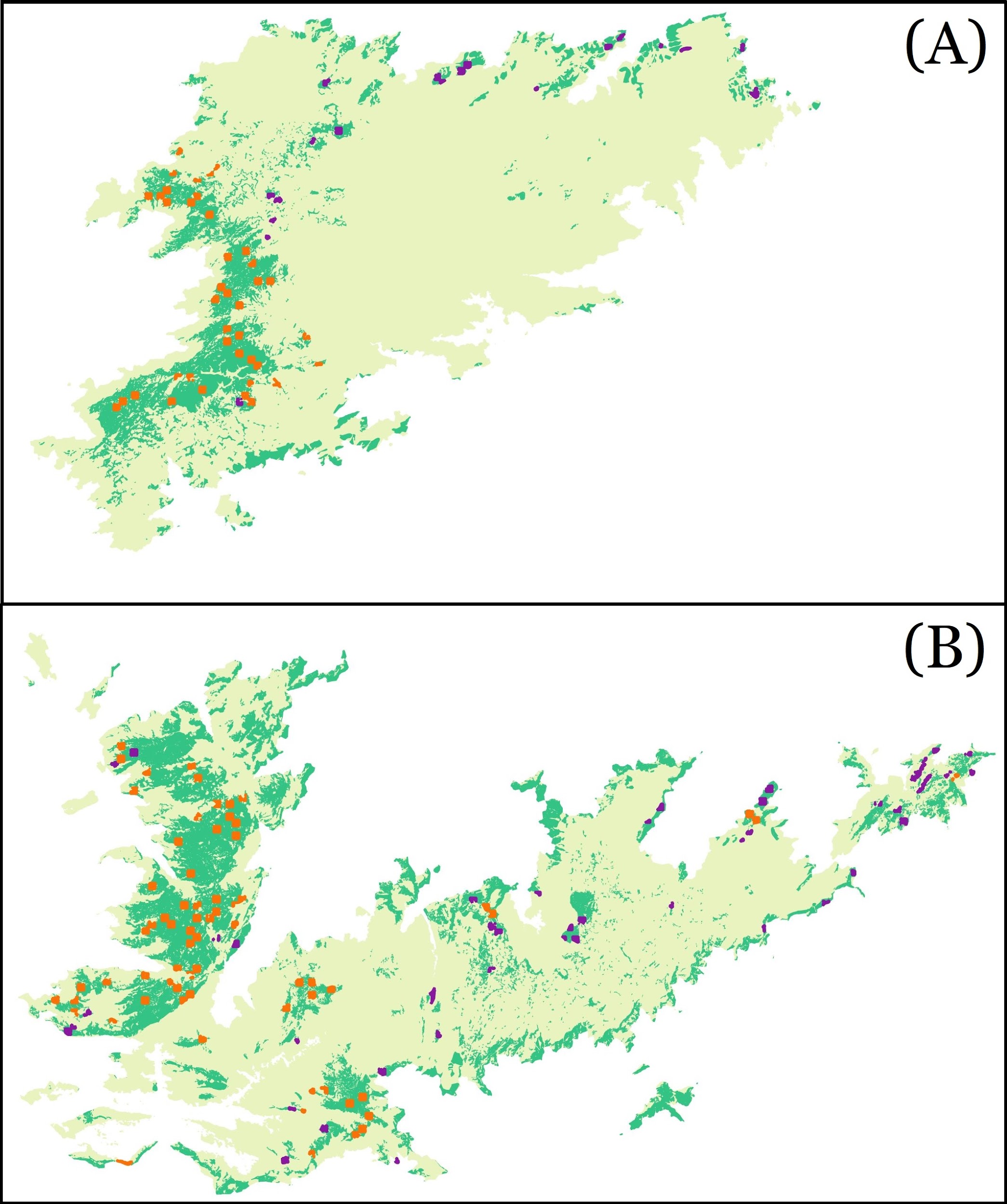


**Supplementary Figure 1.** Detections of the Nilgiri pipit across all sampling locations in the Nilgiris (A) and Annamalai/Palani hills (B). Off-white outline shows the 1600m elevation contour; dark green depicts grasslands; sampling sites with Nilgiri pipit detections shown in orange; those without, in purple.

**10.5 Estimated effects of all putative covariates on occupancy**

**
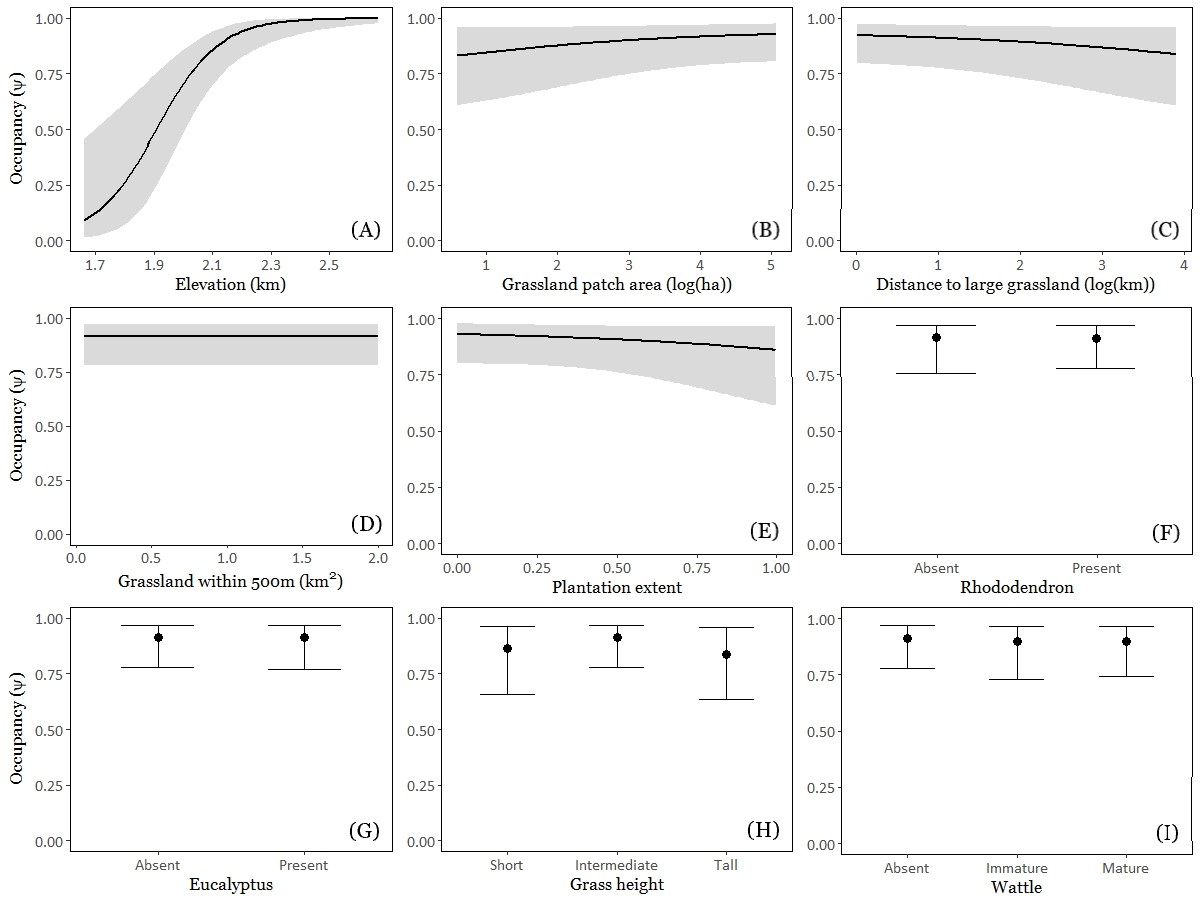
**

**Supplementary Figure 2**. A-I: Model-averaged predicted occupancy of the Nilgiri pipit in response to all covariates appearing in models; maximum elevation within a site (A), log(grassland patch area) (B), log(distance to the nearest grassland larger than 1.5 km^2^) (C), grassland within 500m of the site boundary (D), extent of plantation within the site (E), presence of rhododendron (F), presence of *Eucalyptus* (G), grass height (H), and wattle maturity (I). All other variables are set to mean or modal values for continuous and categorical covariates, respectively. Probability of occupancy is plotted over the observed range of values of each predictor. Bands and error bars represent 95% confidence intervals.

**10.6 Analysis of sites with elevation greater than 1800m a.s.l**

**
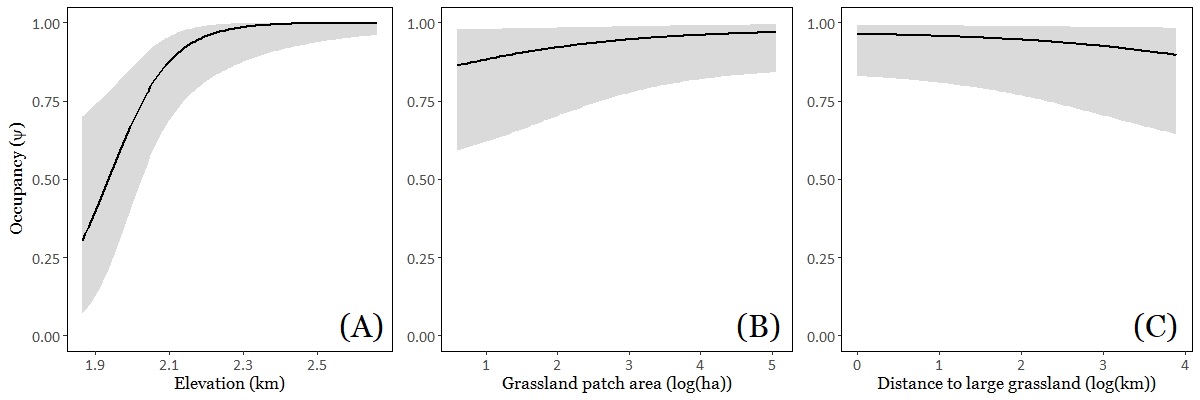
**

**Supplementary figure 3**. A-C: Model-averaged predicted occupancy of the Nilgiri pipit in sites with maximum elevation above 1800m in response to the three covariates with the largest effects; maximum elevation within a site (A), log (grassland patch area) (B), and log (distance to the nearest grassland larger than 1.5 km^2^) (C). All other variables are set to mean values. Predicted probability of occupancy is plotted over the observed range of values of each predictor. Bands represent 95% confidence intervals.

**Supplementary Table 2**: Estimated *β* coefficients for each predictor of occupancy from models with AIC weight ≥ 0.02 for sites with maximum elevation greater than 1800m a.s.l.. In each model presented below, detectability was modelled as a function of (Weather + Day + Grass height + Wattle maturity + Eucalyptus + Rhododendron + Water + Burn + Grassland within 500m). Variable abbreviations are provided in Table 1.

| Occupancy structure | β  MXELEV | β  PCHSZ | β  LGSEP | β  WTMAT  Imm. Mat. | | β  EUC | β  PLEXT | β  RHODO | β  GH  Int. Tall | | AIC | ∆AIC | AIC  Weight | Cum. AIC weight |
| --- | --- | --- | --- | --- | --- | --- | --- | --- | --- | --- | --- | --- | --- | --- |
| MXELEV+PCHSZ | 11.39  ±3.59 | 0.703  ±0.278 | -0.570  ±0.266 |  |  |  |  |  |  |  | 518 | 0 | 0.190 | 0.190 |
| MXELEV+LGSEP | 12.83  ±4.83 |  | -0.732  ±0.317 |  |  |  |  |  |  |  | 518 | 0.24 | 0.169 | 0.360 |
| MXELEV+LGSEP  +PLEXT | 12.66  ±4.18 |  | -0.631  ±0.331 |  |  |  | -1.31  ±1.48 |  |  |  | 519 | 1.59 | 0.0862 | 0.446 |
| MXELEV+PCHSZ  +PLEXT | 11.61  ±3.57 | 0.621  ±0.329 |  |  |  |  | -0.712  ±1.56 |  |  |  | 519 | 1.80 | 0.0775 | 0.523 |
| MXELEV+PCHSZ  +GH+RHODO | 10.15  ±4.33 | 0.620  ±0.248 |  |  |  |  |  | 0.249  ±1.07 | 2.32  ±1.10 | 0.949  ±2.21 | 520 | 2.28 | 0.0609 | 0.584 |
| MXELEV  +WATMAT | 18.38  ±7.09 |  |  | -3.54  ±2.04 | -2.53  ±1.34 |  |  |  |  |  | 520 | 2.47 | 0.0553 | 0.640 |
| MXELEV+GH  +PLEXT | 11.12  ±3.02 |  |  |  |  |  |  |  | 2.67  ±1.08 | 0.783  ±1.60 | 521 | 2.53 | 0.0536 | 0.693 |
| MXELEV | 12.45  ±4.58 |  |  |  |  |  | -2.31  ±1.07 |  | 1.78  ±1.09 | -0.44  ±1.48 | 521 | 2.99 | 0.0427 | 0.736 |
| MXELEV+PLEXT | 12.35  ±3.68 |  |  |  |  |  | -2.20  ±1.32 |  |  |  | 521 | 3.15 | 0.0394 | 0.775 |
| MXELEV+PCHSZ  +PLEXT  +RHODO+GH | 10.49  ±4.45 | 0.497  ±0.310 |  |  |  |  | -0.896  ±1.42 | -0.189  ±1.10 | 2.33  ±1.13 | 0.901  ±1.77 | 522 | 3.88 | 0.0273 | 0.803 |
| MXELEV+LGSEP  +GH+RHODO | 10.49  ±4.47 |  | -0.590  ±0.277 |  |  |  |  | -0.171  ±1.08 | 2.28  ±1.19 | -0.086  ±1.74 | 522 | 3.95 | 0.0264 | 0.829 |
| MXELEV  +EUC | 11.07  ±4.03 |  |  |  |  | -0.839  ±0.944 |  |  |  |  | 522 | 4.30 | 0.0222 | 0.851 |
| MXELEV+LGSEP  +GH+PLEXT  +RHODO | 10.95  ±4.89 |  | -0.440  ±0.308 |  |  |  | -1.57  ±1.26 |  | 2.23  ±1.24 | 0.261  ±1.77 | 522 | 4.42 | 0.0209 | 0.872 |
